# Formulation and characterization of eprosartan mesylate and β-cyclodextrin inclusion complex prepared by microwave technology

**DOI:** 10.1080/10717544.2022.2072540

**Published:** 2022-05-13

**Authors:** Abdul Ahad, Yousef A. Bin Jardan, Mohd. Zaheen Hassan, Mohammad Raish, Ajaz Ahmad, Abdullah M. Al-Mohizea, Fahad I. Al-Jenoobi

**Affiliations:** a Department of Pharmaceutics, College of Pharmacy, King Saud University, Riyadh, Saudi Arabia; b Department of Pharmaceutical Chemistry, College of Pharmacy, King Khalid University, Abha, Saudi Arabia; c Department of Clinical Pharmacy, College of Pharmacy, King Saud University, Riyadh, Saudi Arabia

**Keywords:** Cyclodextrins, docking, inclusion complex, solubility

## Abstract

The goal of this work was to improve the aqueous solubility and dissolution rate of eprosartan mesylate by preparing inclusion complex of drug with β-cyclodextrin (β-CD) by microwave technique. In order to determine the solubility of eprosartan, phase solubility was determined and dissolution study was also conducted. Further, analytical techniques for instance, particle size distribution, differential scanning calorimetry, powder X-ray diffraction, scanning electron microscopy, Fourier-transform infrared spectroscopy, and nuclear magnetic resonance spectroscopy were used for the characterization of inclusion complex. In addition, the binding pattern of eprosartan with the β-CD was investigated by molecular modeling study. Phase solubility study revealed that approximately 4.48 folds improvement in the solubility of drug was noted with β-CD (10 mM). The estimated stability constant (*K*_c_) values for eprosartan:β-CD binary mixture was found to be 280.78 M^–1^. The prepared inclusion complex of drug with β-CD presented better drug release profile (62.96 ± 2.01% in 1 h) as compared to their physical mixture (41.41 ± 1.77% in 1 h) or drug per se (29.97 ± 3.13%). The inclusion complex demonstrated different features and properties from pure drug, and we inferred that this could be due to the inclusion of drug into cyclodextrin cavity that confirmed by different analytical method. Molecular modeling study demonstrated a good affinity of eprosartan to entangle to β-CD. The outcomes have shown that guest molecule has many significant interactions with the host molecule. These observations are very interesting and may be a valuable approach to improve the solubility and in turn the bioavailability of eprosartan.

## 1. Introduction

**Figure 1.**
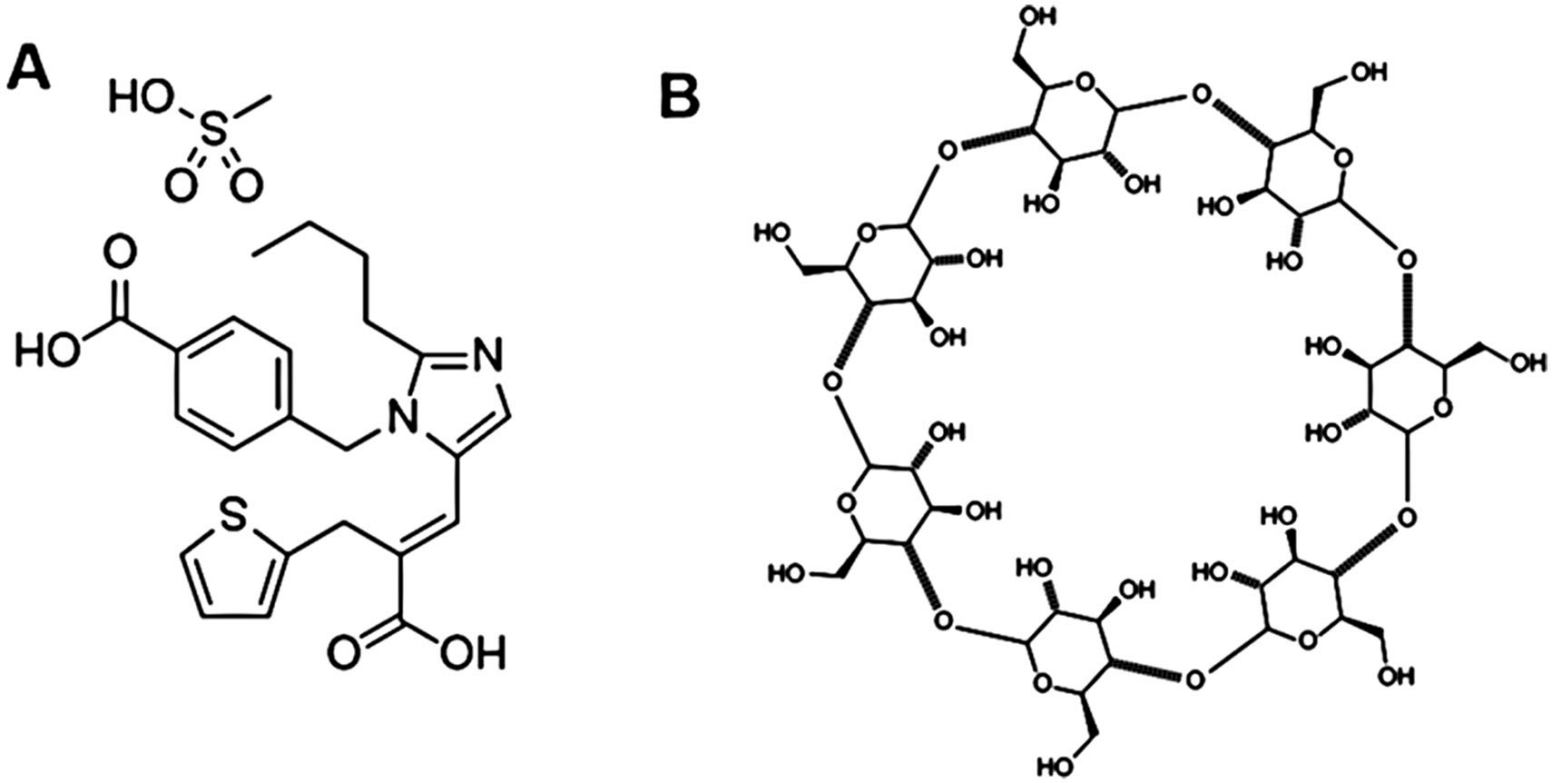
Chemical structure of (A) EM and (B) β-CD.

Eprosartan mesylate (EM, Figure 1(A)) is considered a promising angiotensin II receptor antagonist (Ahad et al., 2018). EM is licensed for the management of hypertension in some more than 20 countries including the UK, Germany, and USA (Ahn et al., 2011).

EM is a medicine that has a low water solubility and is recognized as a class II drug as per Biopharmaceutics Classification System (Ahad et al., 2016). Patients with varying severity of hypertension are commonly given 400–800 mg of EM once or two times everyday (Ahad et al., 2017).

Antecedently, the pharmacokinetic of EM has been acquitted in healthy volunteers. The bioavailability of 13% was found in healthy volunteers and peak plasma concentration of EM was noticed at 1–2 h after an oral dose in the fasted state. In addition to it, the elimination half-life of 5–9 h of EM was observed after oral administration (Tenero et al., 1998; Bottorff & Tenero, 1999). The lower absolute bioavailability of EM after oral administration is substantially lower (∼13%), possibly correlated to low drug absorption rather than to high first-pass elimination. Further, EM revealed a pH-dependent aqueous solubility and lipophilicity, resulting in inconstant absorption as it moves through the digestive tract. Since the oral bioavailability was found only 13%, hence a high dose of drug may be required for an efficient management of hypertension. Since the oral bioavailability of EM is circumscribed by the solubility, hence the improvement in the EM aqueous solubility could advance its oral efficaciousness and lessening the need for such high doses (Yousaf et al., 2018). Accordingly, there is a demand for a formulation that increases the bioavailability of EM. The oral bioavailability of BCS class 2 drug could be increased by improving its aqueous solubility by applying a solubility-enhancing procedure for instance inclusion complex (Alshehri et al., 2020).

Cyclodextrins (CDs) are cyclic oligosaccharides, comprising a least possible of six d-(+)-glucopyranose units bonded by α-1, 4-linkages formed by the process of the CD-trans-glycosidase enzyme on a medium comprising starch. It has a hydrophobic central cavity and a hydrophilic outer surface (Patel & Patel, 2009; Ding et al., 2020; Zhu et al., 2021). CDs have been discovered to be exceedingly beneficial in increasing the solubility of poorly water-soluble drugs due to the establishment of inclusion complex of the drug in its hydrophobic cavity (Ammar et al., 1995; Sathigari et al., 2009; Giri et al., 2021). The utmost common natural CDs are α-cyclodextrin, β-cyclodextrin (β-CD, Figure 1(B)), and γ-cyclodextrin, they are contained six, seven, and eight glucose units, respectively. Various others CDs, derivatives are also investigated (Guo et al., 2013; Suvarna et al., 2017; Mashaqbeh et al., 2021) which could give rise to better drug solubility on complexations (Linares et al., 2000), but monetary value and noxiousness issues present constraint in their application. Amid the numerous available CDs, β-CD are the inexpensive and are harmless for oral use (Redenti et al., 2001; Hirlekar & Kadam, 2009). As CDs are comparatively more water-soluble and EM could establish inclusion complex with CD, and that can formed complex could improve the solubility of the attached EM (Quader et al., 2017; Yousaf et al., 2018). All the above information intensely encourages us to study the possibility of using β-CD as a carrier to ameliorate the solubility of poorly water soluble drug EM. Hence, the aim of the present investigation was to prepare EM:β-CD binary complex with the objectives to increase the solubility and dissolution of the drug. Lastly, the selected EM:β-CD complex was characterized for solid state characterization, and molecular docking.

## 2. Materials and methods

### 2.1. Materials

EM was obtained from BASF (Ludwigshafen, Germany). β-CD was procured from Sigma-Aldrich (St. Louis, MO). Chromafil^®^Xtra filters were purchased from Macherey-Nagel Gmbh & Co. Kg (Duren, Germany). Ethanol was purchased from Avonchem (Macclesfield, UK). All other materials are of analytical grade.

### 2.2. Methods

#### 2.2.1. Phase solubility study

In this study, the excess quantity of EM was taken and transferred to different flask having different β-CD (1–10 mM) solution in water. Likewise, an aqueous suspension of EM per se was also prepared in another flask. Later, all flasks carrying samples were sealed and placed in shaking water bath (model 1083; GFL GmbH, Burgwedel, Germany) for 72 h at 25.0 ± 1 °C and these mixtures were sonicated for 15 min at every 12 h. In completion of 72 h, samples were taken out and supernatant was pipetted out from each sample, filtered and then EM content was ascertained at *λ*_max_ 234 nm using a UV visible spectrophotometer (Ghule et al., 2015).

The stability constant (*K*_c_) for binary sample was estimated from the slope of the phase solubility diagram (Talegaonkar et al., 2007; Ajit Shankarrao et al., 2010; Maeda et al., 2015; Pal et al., 2020). While the solubility of the drug in absence of β-CD (*S*_0_) was accounted by the following equation:

(1) $$K_{c}\;=\;\frac{\mathrm{Slope}}{\mathrm{Intercept}\;(1-\mathrm{Slope})}$$

On the other hand, the extent of formation of inclusion complex of β-CD with EM might be revealed by complexation efficiency (CE) value. Consequently, it is important to ascertained CE value addition to *K*_c_ value and CE was accounted by the following equation (Brewster et al., 2008; Maeda et al., 2015):

(2) $$\mathrm{CE}=\;S_{0}K_{\text{c }}$$

#### 2.2.2. Preparation of inclusion complex

The binary (EM:β-CD, 1:1) inclusion complex was formulated by physical mixing (PM), and microwave irradiation method.

#### 2.2.3. Physical mixing

In this method, binary PM was prepared by mixing of EM:β-CD. The PM each component was weighed accurately and mixed properly to formulate binary (EM:β-CD) mixture.

#### 2.2.4. Microwave irradiation

In this method, the previously estimated quantities of each components of binary (EM:β-CD) were precisely weighed and then the components of binary mixture were properly blended to prepare a homogenous paste in a mortar with the help of pestle using solvent that contained ethanol and water. Each sample of binary mixture was transferred to separate beaker and each beaker (one by one) was placed in a microwave for irradiation (500 W; microwave oven; Samsung model ME0113M1, Seoul, South Korea) (Moneghini et al., 2008). The irradiated sample was agitated to bring a homogenous mass of inclusion complex of EM and β-CD. The formulated inclusion complex of drug and β-CD are gathered and set aside to cool down and stored in desiccator for two days in order to remove any remaining moisture (Moneghini et al., 2009).

#### 2.2.5. Dissolution study

For the dissolution study, EM per se, PM and inclusion complex sample were placed in the dissolution apparatus containing 900 ml of release media, maintained at 37 ± 0.5 °C and rotated at 50 rpm. Aliquots of 5 ml sample were pipetted out at each time point and replenished with fresh release medium. The pipetted out sample was filtered, diluted, and tested for drug content at 234 nm by UV visible spectrophotometer.

Further, the data obtained were implemented to various release kinetic models for each sample (Bhandaru et al., 2015; Dangre et al., 2016; Yousaf et al., 2018; Reddy, 2019).

#### 2.2.6. Particle size

The particle size analysis of EM inclusion complex was performed with zeta sizer (Malvern Instruments, Worcestershire, UK). The prepared aqueous sample of EM binary inclusion complex was diluted with Milli-Q water (100 times), ultrasonicated and passed by a filter membrane (Chromafil^®^xtra 0.45 µm) prior analyzed at 25 ± 1 °C (Li et al., 2015).

#### 2.2.7. Differential scanning calorimetry (DSC)

For DSC study, 5 mg of samples such as EM per se, PM and prepared drug:β-CD inclusion complex were filled in aluminum pans, sealed and analyzed by DSC instrument from 50 °C to 350 °C at the heating rate of 10 °C/min using Perkin Elmer DSC-8000 (Waltham, MA) instrument.

#### 2.2.8. Powder X-ray diffraction (PXRD)

The polymorphic changes were analyzed by PXRD characterization of all prepared samples. PXRD analysis was executed by Ultima IV Diffractometer (Rigaku Inc., Tokyo, Japan), and PXRD pattern of all samples was analyzed from the 5° to 70° 2-theta range.

#### 2.2.9. Scanning electron microscopy (SEM)

The surface characteristics of EM per se, PM and inclusion complex samples were determined by SEM. Samples were prepared according to the procedure and visualized by SEM (Zeiss EVO LS10; Cambridge, UK) at 15 kV accelerating voltage.

#### 2.2.10. Fourier-transform infrared (FTIR)

The FTIR spectra of EM per se, PM and the prepared inclusion complex were collected between 3500 cm^–1^ and 500 cm^–1^ by Bruker Alpha FTIR spectrometer (Billerica, MA) coupled with the OPUS software. All samples were pulverized using spectroscopic grade KBr powder and then compacted into 1 mm pellets. A blank KBr disk was put-upon as background.

#### 2.2.11. Nuclear magnetic resonance (NMR)

In this study, ^1^H (700 MHz) of EM per se, PM and inclusion complex were evaluated by Bruker NMR spectrophotometer (Billerica, MA) coupled software top spin 3.2 with the goal of identifying how EM's carbon and protons interact with the hydrophobic pocket of β-CD. The NMR bands of EM and inclusion complexes were examined in acetone-*d*_6_ at 25 °C.

#### 2.2.12. Molecular modeling

Docking is a technique that can accurately anticipate one molecule’s intended conformation respect to the another molecule when they are bound to form a stable complex with each other. The assessment of produced configurations was focused primarily on the amount of interactions they formed upon binding with the active site residues. Using Autodock 4.2 and the Chem3D 14.0 package, a molecular modeling investigation of eprosartan with -CD was conducted. The eprosartan complex was produced at the β-CD cavity using Autodock tools version 1.5.6 (www.autodock.scrips.edu).

## 3. Results and discussion

### 3.1. Phase solubility study

**Figure 2.**
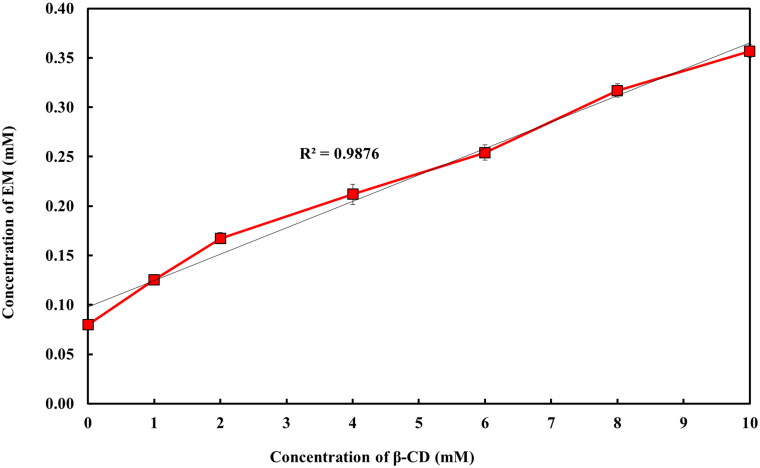
Phase-solubility plot of EM with β-CD.

The outcomes of phase solubility study revealed that the solubility of EM in water was improved consistently on increasing the β-CD concentration in water up to 10 mM (Figure 2) acquiring AL-type solubility diagrams (Higuchi & Connors, 1965). Approximately, 4.48 folds increment in the solubility of EM was observed when β-CD was added at 10 mM as compared to the solubility of EM in water.

In this study, the estimated *K*_c_ and CE values for EM:β-CD binary mixture were found to be 280.78 M^–1^ and 22.43, this *K*_c_ value is considered to be in the range of an ideal value (100–1000 mol L^–1^) and pointing that EM-β-CD complex is appropriately stable (Mukne & Nagarsenker, 2004; De Miranda et al., 2011; Suvarna et al., 2017). In fact, lesser values (below 100 mol L^–1^) of *K*_c_ suggest an excessively weak interaction among the drug and CD and highly unstable complex was formed, whereas greater values (higher than 1000 mol L^–1^) are indicative of an inadequate drug release from the inclusion complex and may hinder the absorption of drugs (Manca et al., 2005; De Miranda et al., 2011; Suvarna et al., 2017).

### 3.2. *In vitro* dissolution study

**Figure 3.**
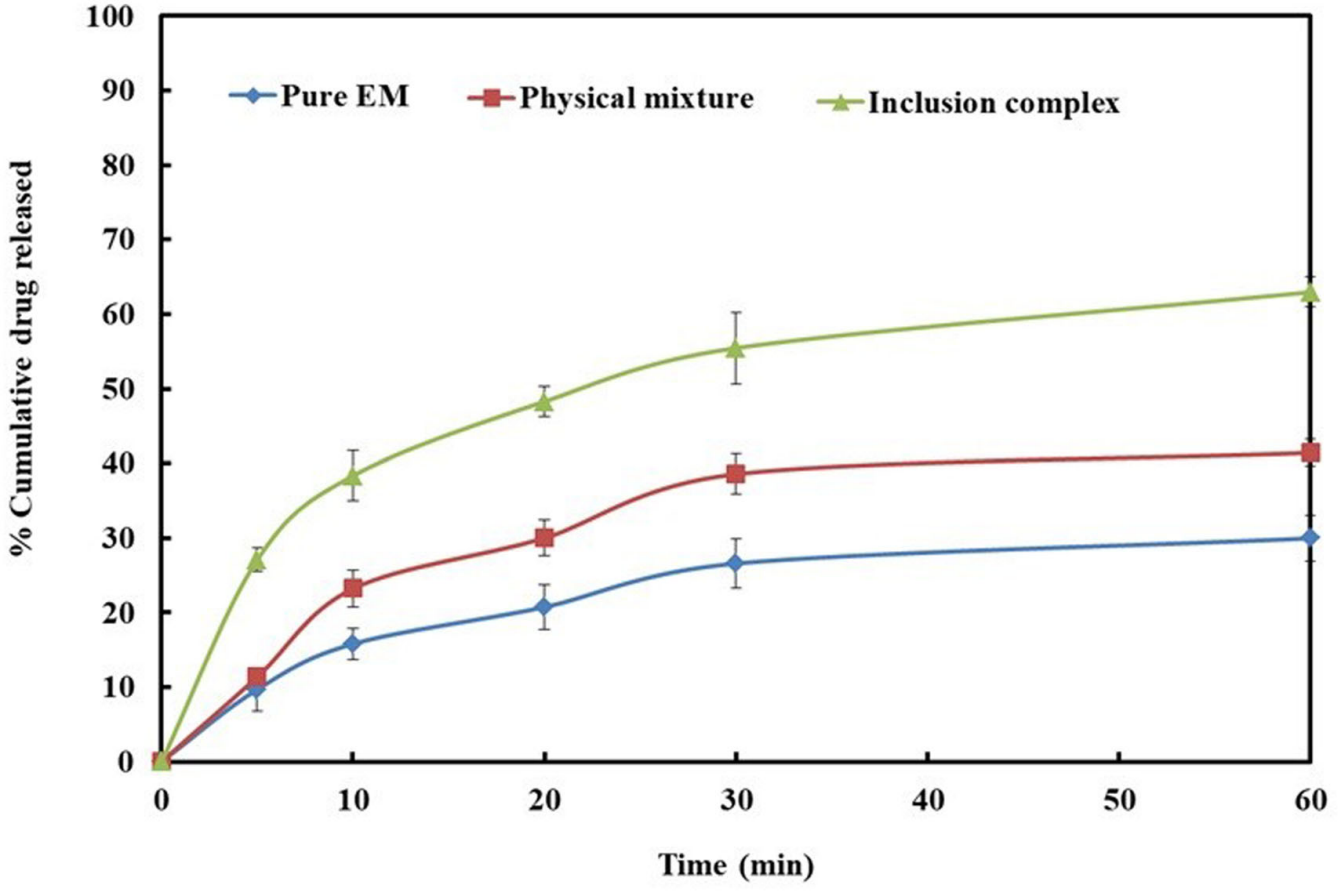
Dissolution profiles of EM per se, physical mixture, and inclusion complex.

In this study, dissolution profile of EM per se demonstrated a low dissolution (29.97 ± 3.13%) during the course of study (Figure 3). It was noted that the pattern of dissolution of EM from its prepared complex and the pattern noted in the phase solubility of EM were approximately similar.

The prepared complex of EM with β-CD has shown improved dissolution profile as compared to their PM (41.41 ± 1.77%) or drug per se (29.97 ± 3.13%). Inclusion complex displayed maximum EM dissolution, i.e. 62.96 ± 2.01% in 1 h (Figure 3). Inclusion complexes formulated by microwave irradiation displayed improvement in the dissolution of EM in comparison to PM, this might be attributed to the greater coupling between the EM, and β-CD by virtue of the energy of microwave irradiation (Badr-Eldin et al., 2013).

The dissolution data so obtained were fitted to the different drug release kinetic models to understand the best possible EM release mechanism from inclusion complex. The release kinetic model which presented highest *r*^2^ (correlation coefficient) value was considered as best drug release model (Costa & Sousa Lobo, 2001; Cascone, 2017). In this study, the release mechanism of the inclusion complex with highest rate and extent was found to be the Peppas type (*r*^2^=0.9677) (Table 1). Further, solid state characterization was done for binary inclusion complex.

**Table 1.** Correlation coefficients calculated by fitting *in vitro* data to different release models.

Release model	Pure EM	Physical mixture	Binary complex
	*R*^2^ value	*R*^2^ value	*R*^2^ value
Zero order	0.8301	0.7482	0.8316
First order	0.8520	0.7864	0.8971
Higuchi’s model	0.9325	0.8739	0.9365
Korsmeyer-Peppas	0.9567	0.8977	0.9677
Hixson-Crowell	0.8449	0.7741	0.8766

### 3.3. Particle size

**Figure 4.**
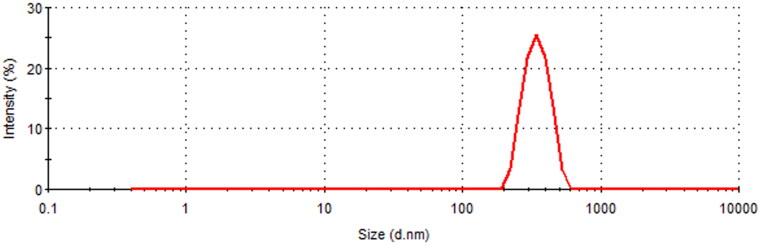
Particle size image of EM-inclusion complex.

The prepared EM–binary complex sample presented the particle size of 537 nm with polydispersity index of 0.386 as displayed in Figure 4.

In our study, the particle size of prepared inclusion complex was found much larger that particle size of pure β-CD reported somewhere else (Li et al., 2015). This could be due to the entrapment of EM into β-CD complex. Further, it was reported that in aqueous solutions, CDs and their complexes may produce large aggregates that are soluble in water and these aggregates could enhance the solubility of poorly soluble drugs (Loftsson et al., 2004).

### 3.4. Differential scanning calorimetry

**Figure 5.**
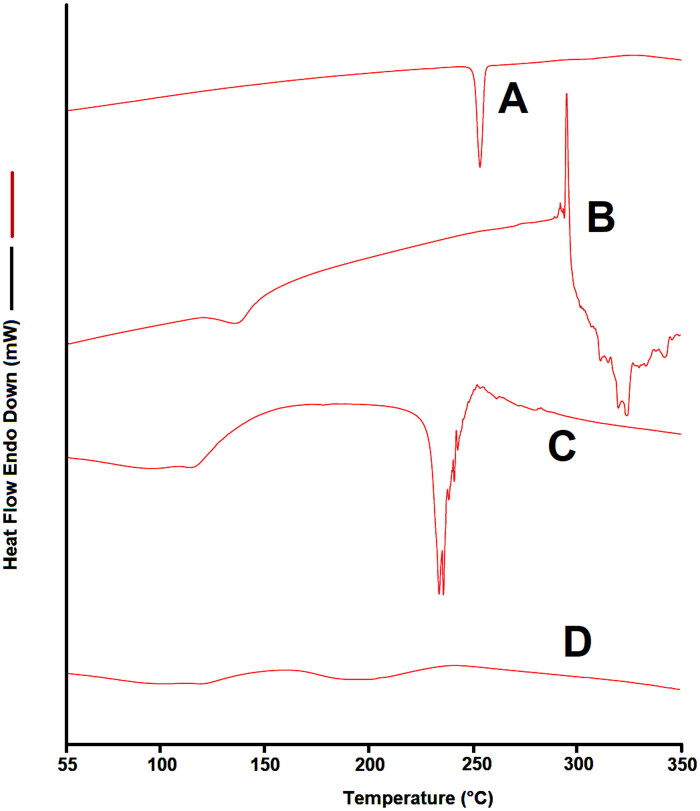
DSC thermogram of (A) EM per se, (B) β-CD per se, (C) physical mixture, and (D) inclusion complex.

Figure 5 displays the DSC thermograms of EM per se, β-CD, PM as well as the complex prepared by microwave irradiation. The DSC image of EM per se displayed a melting endothermic point at about 253.50 °C as demonstrated in Figure 5(A).

While the DSC image of PM displayed the representative peak of EM was apparently distinct in the PM, and there is a little distortion of EM peak is visible, this indicating very week interaction between EM and β-CD which could be occurred in the course of mixing or heating for DSC analysis (Huang et al., 2019). While in the DSC image of sample of inclusion complex, the endothermic peak of EM is completely melted that could be due to the formulation inclusion complex. The complete evaporation of the endothermic EM peak in the prepared complexes proposed that was a firmed interaction among EM and β-CD and hence EM was considerably enclosed in the cavity of β-CD during the complex formation. Our results are in agreement with the earlier report that investigated the effects of CDs on the thermal behavior of drug by DSC; authors noted that CDs could cause rise of new peaks, shifting, broadening, and diminishing of some peaks (Ramnik et al., 2010; Badr-Eldin et al., 2013). In another study, similar circumstances were reported when drug were complexed by β-CD, and the endothermic peak of drug was completely vanished in the inclusion complex (Sharma & Jain, 2013; Huang et al., 2019).

### 3.5. Powder X-ray diffraction

**Figure 6.**
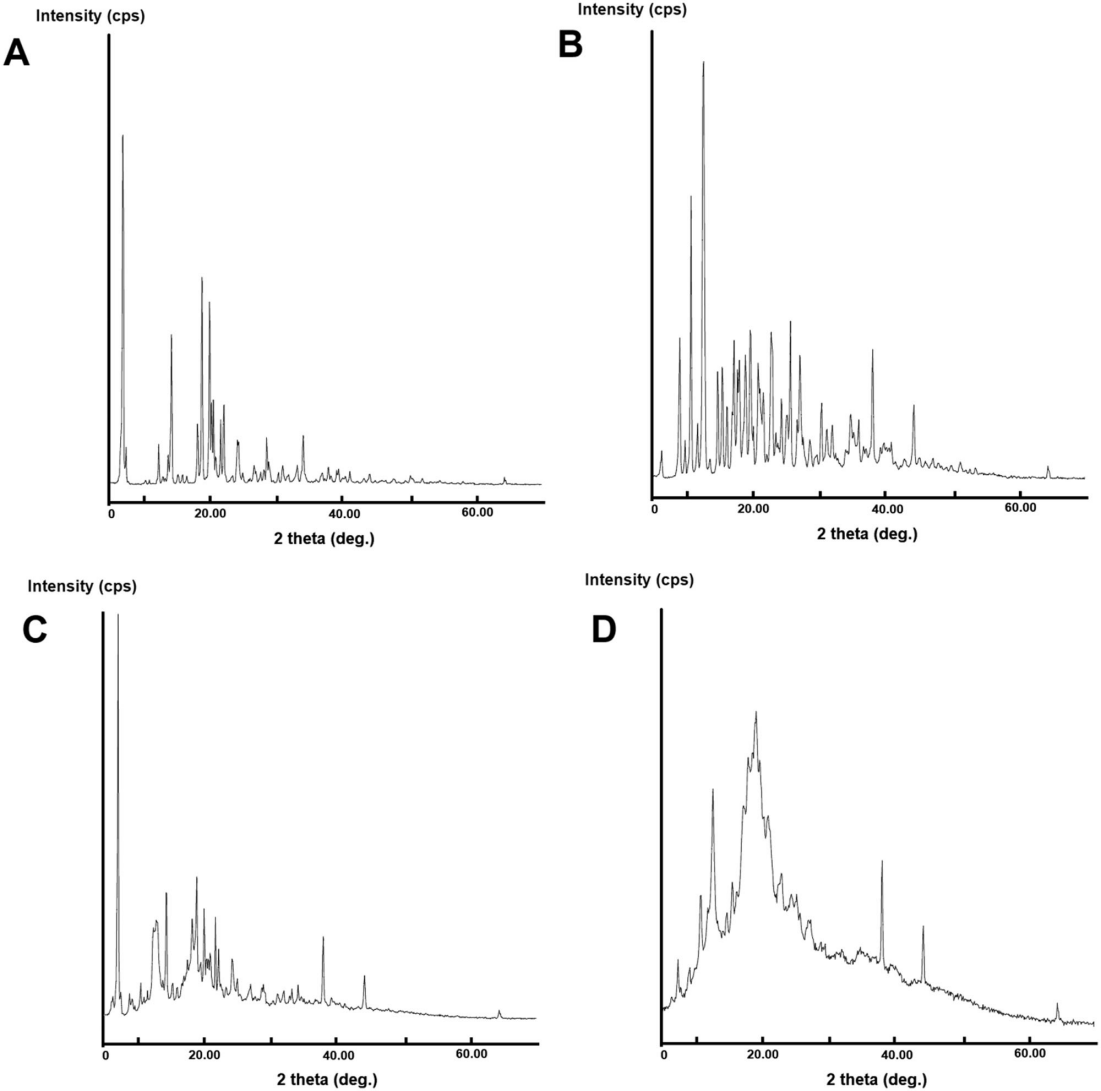
XRD patterns of (A) EM per se, (B) β-CD per se, (C) physical mixture, and (D) inclusion complex.

Another technique employed to ascertain the inclusion complex of drug with β-CD was PXRD analysis (Figure 6).

The PXRD is valuable technique for quick recognition of new crystalline phase in solid state. This technique delivers the evidence for the existent of crystalline form of drugs and discloses the probable organization of atoms within the crystal lattice. The PXRD graph of EM per se showed characteristic peaks to 2*θ* at 14.0, 14.5, 18.4, 19.1, 20.2, 20.5, 20.8, 21.1, 21.9, 22.4, 24.4, 24.6, 26.9, 28.8, 29.1, 31.2, 33.4, 34.3, and 38.0, the results are in agreement with the previous study (Bhandaru et al., 2015).

The presence of numerous strong crystalline peaks reveals the presence of EM in crystalline form. The majority of characteristics peaks of EM are visible in PXRD pattern of PM which supports the purity of the EM in the PM formed suggesting week interaction in the PM (EM:β-CD).

However, the inclusion complex of EM with β-CD exhibited the new diffraction peaks and the reduction of characteristics peaks intensity was also observed. This pointing the strong interaction of EM and β-CD that indicated the formation of a considerable portion of amorphous material of EM in the binary complex (Figure 6(D)).

### 3.6. Scanning electron microscopy

**Figure 7.**
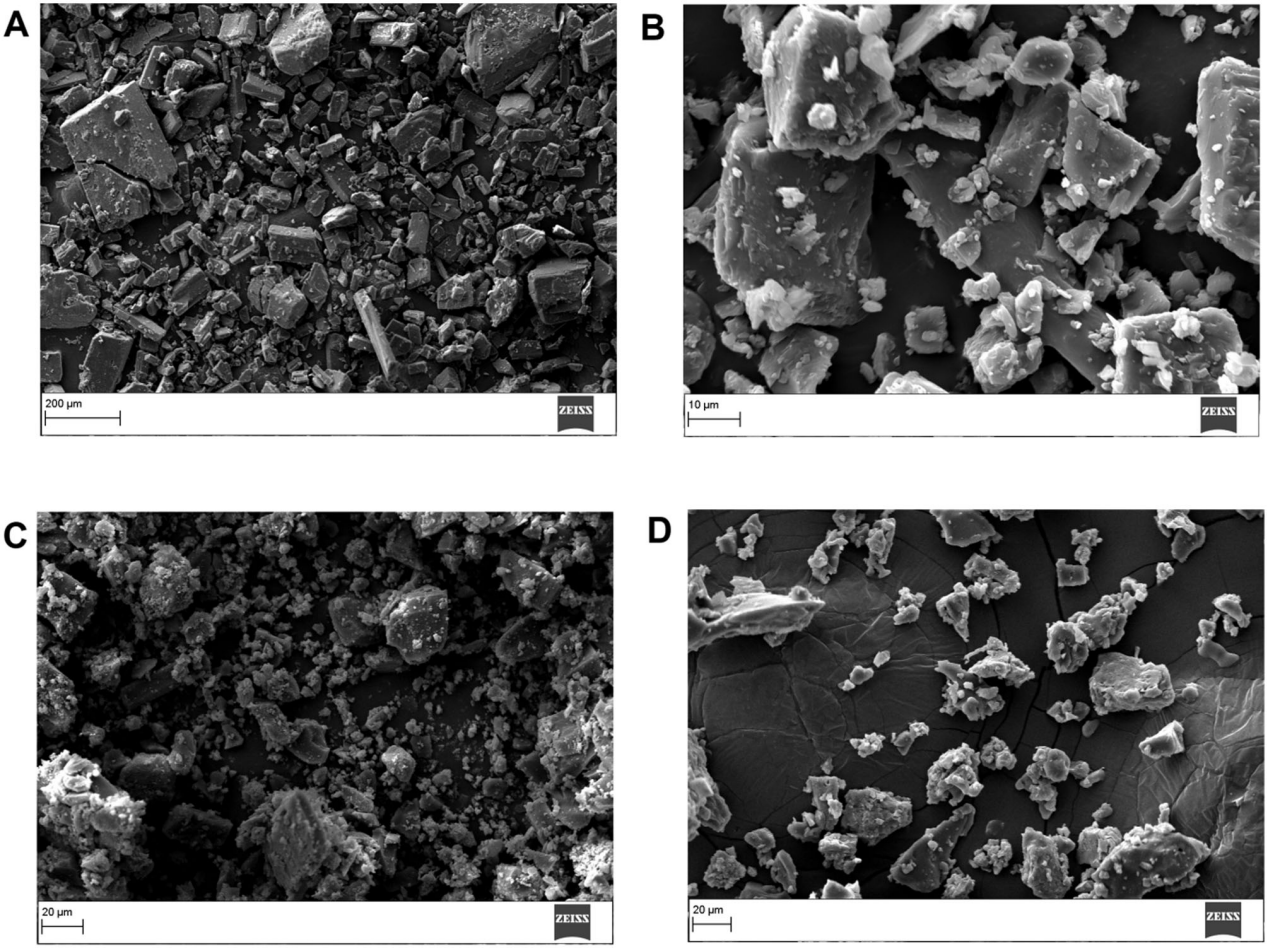
SEM image of (A) EM per se, (B) β-CD per se, (C) physical mixture, and (D) inclusion complex.

The surface morphology of powder derived from EM per se, PM and inclusion complex was examined by SEM (Figure 7).

SEM image of EM per se displayed as rod shaped crystals featuring a rough surface (Figure 7(A)) (Dangre et al., 2016). The β-CD consisted of cluster structure has a globular shape particle with irregular sizes (Alshehri et al., 2020). While in the SEM scan of PM, distinctive EM crystals, which were mingled with β-CD crystals or cohered to their surface, were apparently discovered (Figure 7(C)). On the other hand, in the EM/β-CD inclusion complex (Figure 7(D)) displayed as irregular particles in which the characteristic surface morphology of EM and β-CD melted and reduced size amorphous particles having irregular size and shape were clearly visible. Inclusion complex image demonstrated that the particles of the inclusion complex were physically different from the morphology of individual excipients and their PM, which have supported the formation of the inclusion complex (Naidu et al., 2004).

### 3.7. Fourier-transform infrared spectroscopy

**Figure 8.**
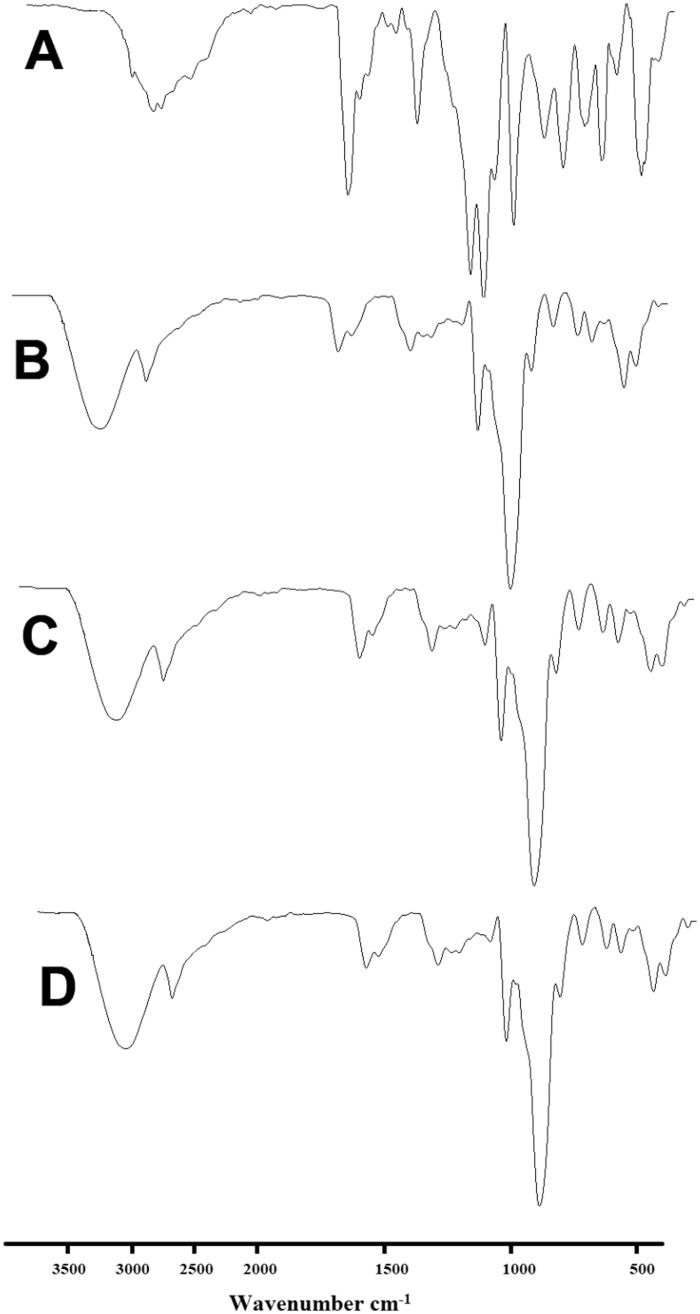
FTIR spectra of (A) EM per se, (B) β-CD per se, (C) physical mixture, and (D) inclusion complex.

The FTIR spectrum of EM per se displayed the characteristic peaks at 534 cm^–1^, 691 cm^–1^, 758 cm^–1^, 844 cm^–1^, 1039 cm^–1^, 1159 cm^–1^, 1210 cm^–1^, 1421 cm^–1^, 1696 cm^–1^, and 2926 cm^–1^. The peak corresponding to CO, SO_2_, and O–H group is found at 1696 cm^–1^, 1210 cm^–1^, and 1421, respectively (Figure 8(A)). These peaks are in agreement with the previously reports (Bhandaru et al., 2015; Dangre et al., 2016; Yousaf et al., 2018; Shekhawat & Pokharkar, 2019; Tekko et al., 2019).

The FTIR spectra of β-CD per se presented bands between 2923 cm^–1^ and 3284 cm^–1^ conforming the presence of OH groups, band appeared at 1702 cm^–1^ could be owing presence of H_2_O in the cavity, and a large peak that showed individual bans between 1021 cm^–1^ and 1149 cm^–1^, could be accountable for the presence of C–O vibrations (Sharma & Jain, 2013). These FTIR bands are clearly visible in the sample of PM (Figure 8(C)). While the intensity and position of these peaks were altered radically in FTIR patters of prepared inclusion complex in comparison to the FTIR patter of EM per se and PM. This pointed that the vibrating and bending of the EM (guest molecule) was constrained owing to the establishment of an inclusion complex, therefore, it is certainly possible that the aromatic rings in EM were enclosed into the cavity of β-CD (Wen et al., 2004).

### 3.8. Nuclear magnetic resonance spectroscopy

**Figure 9.**
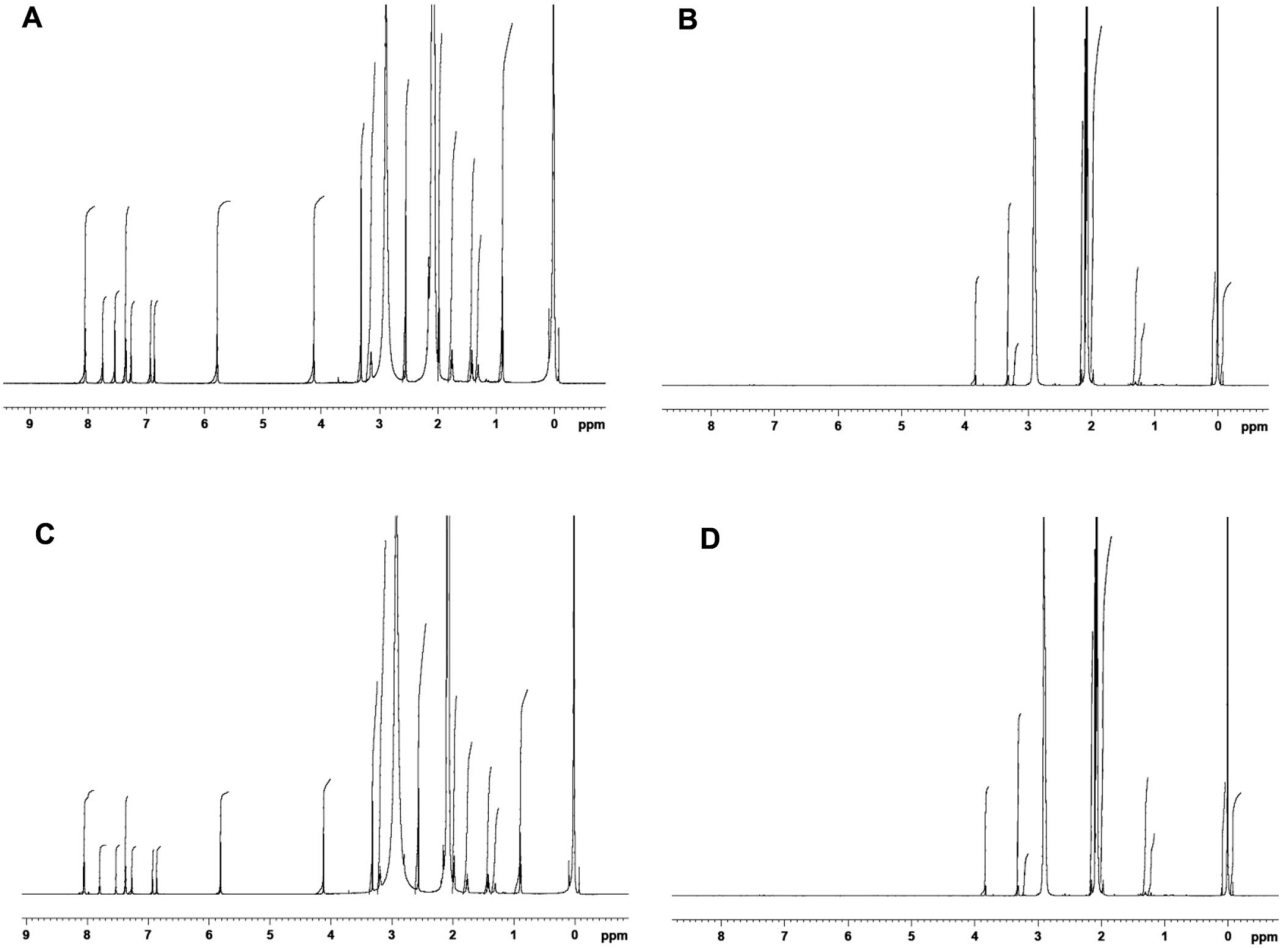
^1^H NMR spectra of (A) EM per se, (B) β-CD per se, (C) physical mixture, and (D) inclusion complex.

The ^1^H NMR spectra of EM per se, β-CD per se, PM, and binary complex, are demonstrated in Figure 9.

This study examines the interactions among the EM per se and β-CD by means of chemical shift (*δ*), as illustrated in Figure 9(A,B). The ^1^H NMR spectra of EM per se in acetone-*d*_6_ revealed a noticeable deshielded singlet at 8.06 ppm, this could be due to presence of carboxylic proton. The aromatic 4H carbon at C-8, 9, 11, and 12 demonstrated the multiple peaks at 7.26–7.36, and a singlet peak was noted for methylene at position 15, with a *δ* value of 3.32 ppm. The ethylene peak at C-13 was noted at 7.75 ppm.

In addition to this, NMR spectra also displayed one intense peak at *δ* 5.78 ppm, this could attribute to the presence of imidazole ring however multiplet peaks at *δ* 6.86–6.94 ppm could be due to the presence of thiophene. The structure of EM also indicated singlet peak of methylene at C-6 expressing *δ* value of 4.99 ppm. Lastly, the butyl group presented multiplet peaks at *δ* 1.31–2.01 ppm. On the other hand, the PM and binary complex presented little variations in the chemical shift values of aromatic rings, with a multiplet *δ* value of 7.26–7.38 ppm and a singlet peak of *δ* 7.80 and 7.74 ppm, for ethylene at C-13, respectively. A slight alteration was noted for the imidazole peak at *δ* values of 5.81 for PM sample and 5.77 ppm for EM–binary complex. Further, a little alteration was also noted for butyl group having multiplet peaks at *δ* 1.31–2.01 ppm for both samples (PM and binary complex). The ^1^H NMR results of EM–binary complex exhibited a major change in peaks demonstrating its importance in solubility enhancement. The singlet peak at *δ* 2.91 ppm for the hydroxyl group of β-CD was also present in complex with a slight deviation at 2.93 ppm. The outcomes of NMR study support the role of –OH group of β-CD in complex making with drug which consequences the enhancement of solubility of EM. Additionally, the aromatic protons of EM displayed a substantial up-field shift; this could be due to the presence of β-CD. Further, the existence of β-CD glucose peak at *δ* 3.83 ppm was also discovered in complex with slight deviation in *δ* values at 3.71 ppm; this chemical shift also further established the formation of complex.

### 3.9. Molecular modeling

**Figure 10.**
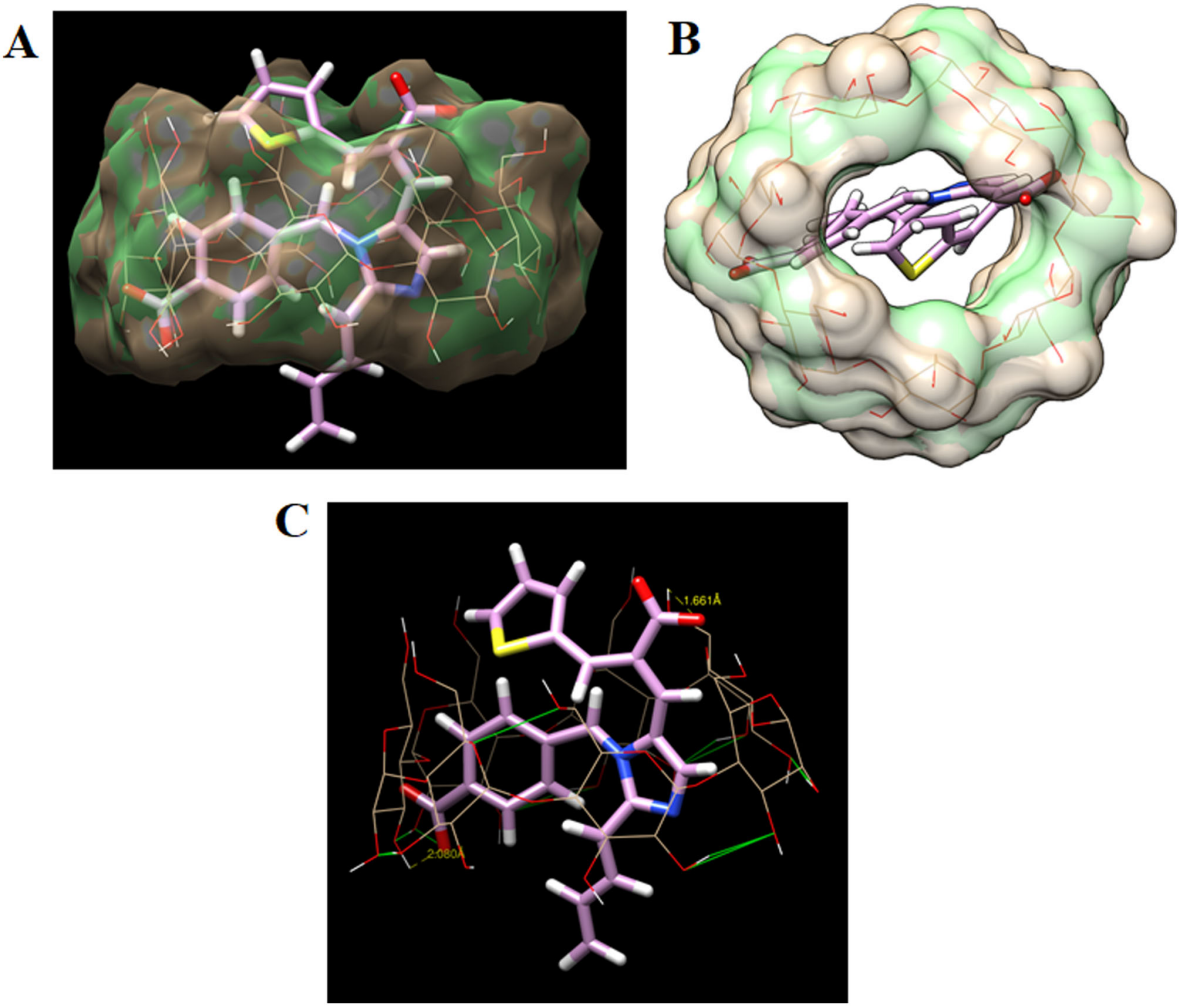
(A) Surface view showing binding configuration of host–guest complex showing eprosartan (stick) inside the cavity of β-CD, (B) surface view showing centrally occupied eprosartan (stick) in the central cavity of β-CD, and (C) detailed binding interactions of eprosartan (stick) at the central cavity of β-CD (wire) showing two hydrogen bonds (dotted yellow line).

The docking approach was utilized to examine EM's attachment behavior with the β-CD. To delve deeper into this aspect, we undertake molecular docking of eprosartan into the cavity of β-CD. The docked conformations of the ligand eprosartan bound to the β-CD are shown in Figure 10. The central less hydrophilic toroid of β-CD well accommodated the eprosartan. The docking investigation has disclosed that the affinity for binding the eprosartan at the β-CD was −6.2 kcal/mol, this strongly demonstrates the complex's stability. The binding configurations of drug with β-CD disclosed that the eprosartan inhabited the central region of β-CD by creating two hydrogen bonds. The 2-carboxy group of eprosartan made a tight hydrogen connection with the primary hydroxyl group of glucopyranose in β-CD, with the bond length of 1.66 Å. The second hydrogen bond was observed between the benzoic carboxy group and secondary hydroxyl group of glucopyranose (2.08 Å). Aromatic benzene ring and imidazolyl ring of eprosartan inhabited the hydrophobic region of β-CD which established multiple CH–π connections; however, the aliphatic butyl group formed hydrophobic contacts with the glucopyranose.

## 4. Conclusions

The complex inclusion of EM was successfully prepared by microwave irradiation method. It was concluded the solubility of EM was substantially increased in presence of β-CD, in addition to this, binary complex showed improved solubility of EM than PM; hence, we can concluded that the microwave technology substantially improved the complexation proficiency of EM and β-CD and consequently ameliorated the solubility of EM in binary complex. Solid characterization for instance, DSC, FTIR, PXRD, SEM, and NMR analysis endorse the formation of inclusion complex. Furthermore, the *in vitro* dissolution tests recommended that the binary complex was further enhanced the *in vitro* dissolution profile of EM. Moreover, the docking outcomes validate that the investigated drug is entirely encapsulated into the β-CD structure and hence, better solubility was observed with sample prepared by microwave irradiation method as compared to the EM per se.
